# MSCs helped reduce scarring in the cornea after fungal infection when combined with anti-fungal treatment

**DOI:** 10.1186/s12886-019-1235-6

**Published:** 2019-11-14

**Authors:** Yue Zhou, Yuqing Chen, Suiyue Wang, Fangyuan Qin, Liya Wang

**Affiliations:** 1 Department of Ophthalmology, The First Affiliated Hospital of Zhengzhou University, Zhengzhou, 450003 People’s Republic of China; 2 Henan Provincial People’s Hospital, Henan Eye Hospital, Henan Eye Institute, People’s Hospital of Zhengzhou University, Zhengzhou, 450003 People’s Republic of China

**Keywords:** Fungal keratitis, Corneal scar formation, Umbilical cord mesenchymal stem cells

## Abstract

**Background:**

Fungal Keratitis (FK) is an infective keratopathy with extremely high blindness rate. The damaging effect of this disease is not only the destruction of corneal tissue during fungal infection, but also the cornea scar formed during the healing period after infection control, which can also seriously affect a patient’s vision. The purpose of the study was to observe the effect of umbilical cord mesenchymal stem cells (uMSCs) on corneal scar formation in FK.

**Methods:**

The FK mouse model was made according to a previously reported method. Natamycin eye drops were used for antifungal treatment 24 h after modeling. There are four groups involved in the study, including control group, FK group, vehicle^inj^ FK group and uMSCs^inj^ FK group. Mice in uMSCs^inj^ FK group received repeated subconjunctival injections of uMSCs for 3 times at the 1d, 4d and 7d after FK modeling. At 14d, 21d and 28d after trauma, clinical observation, histological examination, second harmonic generation and molecular assays were performed.

**Results:**

The uMSCs topical administration reduced corneal scar formation area and corneal opacity, accompanying with decreased corneal thickness and inflammatory cell infiltration, following down-regulated fibrotic-related factors α-SMA, TGFβ1, CTGF, and COLI and finally inhibited phosphorylation of TGFβ1/Smad2 signaling pathway during FK corneal fibrosis.

**Conclusion:**

The results confirmed that uMSCs can improve corneal opacity during the scar formation stage of FK, and exert anti-inflammatory and anti-fibrotic effects.

## Background

Fungal Keratitis (FK) is an infective keratopathy with extremely high blindness rate. The damaging effect of this disease is not only the destruction of corneal tissue by fungus, but also the cornea scar formation during the healing period after infection control, which can also seriously affect the patient’s vision [1]. Previous studies have focused on the killing and elimination of fungi, while less attention has been paid to the formation of corneal scars after infection control. Corneal transplantation is an effective means to treat corneal scars, but the source of corneal donors in China is limited, problems such as immune rejection is difficult to resolve, and there are still a large number of patients unable to get treated.

Umbilical cord mesenchymal stem cells (uMSCs) are derived from the newborn umbilical cord tissue Wharton’s jelly, which exhibit multipotential differentiation potential, low immunogenicity and immunomodulatory effects [2]. The study of uMSCs in ocular surface mostly focuses on corneal epithelial damage [3], corneal transplantation [4], dry eye [5], etc. However, few studies focus on the effects of uMSCs on corneal fibrosis. We conducted this research to explore potential mechanism and optimized utilization of uMSCs therapy in the field of corneal fibrosis induced by infectious eye disease.

## Methods

### Animals

240 male C57BL/6 J male wild-type mice, aged 8–12 weeks, weighing 18-25 g, were purchased from Nanjing University-Nanjing Institute of Biomedical Research (Nanjing, China). The mice were housed in an SPF-class animal laboratory with room temperature 20–25 °C, suitable humidity, automatic feeding water, and day and night natural light. Mice were anesthetized by intraperitoneal injection with 1% sodium pentobarbital, and sacrificed by cervical dislocation. The feeding and disposal of experimental animals in this study was in accordance with ARVO’s statement on the use of animals in ophthalmology and visual science research and was approved by the Medical Ethics Committee of the Henan Provincial Eye Hospital.

### Primary culture and identification of uMSCs

The umbilical cords were taken from a healthy fetus delivered by a maternity cesarean section of the obstetrics department of Henan Provincial People’s Hospital. It was treated within 1 h after aseptic collection. Infectious diseases such as hepatitis B, hepatitis C, syphilis, AIDS, cytomegalovirus and Epstein-Barr virus are excluded by serological testing before maternal surgery. Written informed consent was obtained from the donors. Fresh umbilical cords were washed with 0.01 M pH 7.2~7.4 phosphate buffer saline (PBS) supplemented with antibiotics (100 U/ml of streptomycin, 100 U/ml of penicillin) twice to remove blood. After treated with 70%ethanol, umbilical cords were then minced into small pieces and incubated with DMEM/F12 medium (1:1) (Hyclone Laboratories; Thermo Fisher Scientific Life Sciences, USA) supplemented with 10%fetal bovine serum (FBS; Hyclone Laboratories) in dishes at 37 °C, 5%CO_2_ supplement. Cells were trypsinized and collected for subculture when they reached 80% confluence. Only uMSCs in passages 2–5 were used in our study. Cultured cells were fluorescence marked with specific mensenchymal stem cell surface antigen CD29, CD44, CD34 and CD45 (BD biosciences, New Jersey, US) and identified by flow cytometry.

### FK model preparation

Mice were anesthetized by intraperitoneal injection with 1% sodium pentobarbital. The right eye of each mouse was selected as the experimental eye. The preparation of the model refers to the article published by our research group [6]. Under the operating microscope (Topcon OMS-90, Japanese), a cross scratch in the center of the cornea was made by using a sterile blade (carbon steel, size 11, Shanghai Medical Suture Needle Factory Co., Ltd., China). The scratch depth was to exceed Bowman’s membrane and stop at the corneal stroma superficial layers. A sterile bamboo stick tip (tip diameter 0.30 mm, length 1.10 mm) was used to pick up a small amount of hyphae, and then apply evenly to the scratches. 24 h after injury, antifungal drug natamycin eye drops were used topically to FK mice eyes 6 times per day for 7 days to inhibit fungi growth. The experimental fungus standard strain Fusarium oxysporum (No. 3.791) was purchased from the General Microbiology Center of the China Microbial Culture Collection Management Committee, Institute of Microbiology, Chinese Academy of Sciences, Beijing. After 24 h of modeling, observation under the slit lamp microscope showed that the corneal fungal infection and a small amount of empyema in the anterior chamber formed were successfully modeled. Cases of uninfected were removed, and the number of deletions was substituted by subsequent experiments.

### Experimental grouping

The mice were randomly divided into four groups, including control group, FK group, vehicle^inj^ FK group and uMSCs^inj^ FK group. Control group referred to unwounded ones, whereas FK group referred to wounded ones without any injection but antifungal drug therapy, meanwhile vehicle^inj^ FK group referred to wounded ones with PBS subconjunctival injection and antifungal drug therapy, finally uMSCs^inj^ FK group referred to wounded ones with uMSCs subconjunctival injection and antifungal drug therapy.

### Topical administration of uMSCs

Mice in the uMSCs^inj^ FK group were anesthetized by intraperitoneal injection of pentobarbital sodium. Under the operating microscope, the Hamilton micro-syringe (Hamilton, Switzerland) with a 30G needle on it was used to carefully insert into the conjunctiva sac and slowly injected 5 × 10^4^ uMSCs in 5 μl PBS. The operation was performed for 3 times at the 1d, 4d and 7d after modeling. Others in vehicle^inj^ FK group received a subconjunctive injection of 5 μl PBS at the same time points using the same method. However, control group and FK group received no injection at all.

### Observation and examination

The mice were observed under a slit lamp microscope every other day, and the ocular surface photographs were taken and corneal opacity scores were graded at 14d, 21d, and 28d after modeling. The score was performed by another investigator blinded to the group to minimize the bias. The scoring criteria are as follows: grade 1 (mild corneal haze, pupil iris clearly visible), grade 2 (superficial corneal opacity, visible pupil and iris through the lesion), grade 3 (uneven full-thickness corneal opacity), grade 4 (homogeneous and dense opacity). The area of corneal leukoplakia (mm^2^) was calculated by using the EyeStudio software.

### Pathlogical examination and hematoxylin-eosin (HE) staining

For the purpose of histological analysis, mice were sacrificed by cervical dislocation and eyeballs were dissected at 14d, 21d and 28d after modeling. After a fixation in 4% paraformaldehyde for 24 h, the tissues were dehydrated, dipped in wax, embedded, and sliced in sequence. The slices were under a routine operation of dewaxing and then HE staining was used for pathological examination. Photos were taken by Nikon 80i light microscope (Nikon, Sendai, Japan) and analyzed in terms of corneal thickness.

### Whole mount corneal immunofluoresence staining

The mice were sacrificed by cervical dislocation at 14d post-injury. The intact eyeballs were removed and then fixed in 4%paraformaldehyde for 90 min at 4 °C. Trim the eyeball, retain the cornea and limbus, and remove the iris, ciliary body and other tissues. Corneas were washed with 0.01 M PBS for 5 times (3 min each), and then dipped in 0.2%Triton-2%BSA 1:100 diluted anti-alpha smooth muscle actin antibody (ab5694, Abcam, Cambridge, US) overnight at 4 °C. After conjugated with 1:500 diluted fluorescence-conjugated secondary antibody Alexa488 (A10042, eBioscience, US) in PBS overnight at 4 °C, the corneas were then flatmounted with four radial cut, and sealed with mounting medium. Images were captured by tissue panoramic scanning microscopy (Pannoramic 250/MIDI, 3D HISTECH, Hungary).

### Second harmonic generation (SHG)

At 14d, 21d and 28d post-injury, mice were deeply anesthetized and fixed on a designated plate, with the ocular surface faced up. According to a previously described method [7], a modified plastic bowl containing sterile PBS was fixed on the ocular surface sealed by erythromycin eye ointment to ensure the use of water immersion objective. SHG imaging was performed using an inverted two-photon excitation fluorescence microscope (NLO780, Zeiss). The laser was tuned to 780 nm and a 20× water immersion objective (numerical aperture =1.0) was used to focus the excitation beam and to collect backward signals. The cornea was scanned layer by layer using Z-stack (Z = 5 μm), and the obtained images were three-dimensionally reconstructed using Imaris software (× 64, version 7.4.2, Bitplane, Zurich, Switzerland) to calculate the average signal intensity of the image. The stronger the image signal intensity, the more regular the corneal matrix collagen fibers are arranged, and conversely, the weaker the image signal, the corneal matrix collagen fiber structure is disordered or degraded.

### Quantitative real-time PCR

The mice were sacrificed by cervical dislocation at 14d, 21d and 28d post-injury and the corneas were trimmed as described above. Corneas were cut into small pieces and grinded for RNA extraction. RNeasy Mini Kit (Qiagen, US) was used to extract total RNA according to the manual and cDNA was generated by reverse transcription (TIANScript RT Kit, TRANSGEN BIOTECH, Peking, China). Real-time amplification was performed using TransStart Top Green qPCR Supermix (AQ131, TRANSGEN BIOTECH, Peking, China) in ABI 7500 Real Time PCR System (Applied Biosystems/Life Technologies) for the following molecules: α-Smooth muscle actin (α-SMA), forward sequencing: TCAGACCTGTGTGTTCCCTA, reverse sequencing: AGACGTGCTTCTTTTCCTTG, transforming growth factorβ1 (TGFβ1), forward sequencing: AAGCCCGAGGACCACATTTT, reverse sequencing: GGGGCCTTTGGCTCAGAAAT, connective tissue growth factor (CTGF), forward sequencing: CATCTCCACCCGAGTTACCA, reverse sequencing: TGCACTTTTTGCCCTTCTTA, collagen type I (COLI), forward sequencing: TCAGACCTGTGTGTTCCCTA, reverse sequencing: AGACGTGCTTCTTTTCCTTG, and housekeeping gene β-actin, forward sequencing: GGCACCACACCTTCTAC, reverse sequencing: CTGGGTCATCTTTTCAC. Gene expression was standardized to housekeeping gene expression in respective samples. Each individual experiment was done in triplicate.

### Western blot assay

The mice were sacrificed by cervical dislocation at 14d post-injury and the corneas were trimmed as described above. 0.1 g tissue was fully grinded in liquid nitrogen, and then transferred to 1 ml RIPA protein lysate. Determination of sample protein concentration was by Coomassie Brilliant Blue Kit. Protein separation was performed by electrophoresis in different concentrations of gels. Polyvinylidene fluoride membranes (Bio-Rad Laboratories, Hercules, CA, USA) were used to transfer the samples. The membranes were blocked with a blocking solution (PBST solution containing 5% skim milk powder) for 2 h at room temperature and then incubated in a 1:1000 dilution of primary rabbit polyclonal anti-Smad2 antibody (bs-0718R, Bioss Antibodies, Peking, China) and primary rabbit polyclonal anti-phospho-Smad2 antibody (bs-5618R, Bioss Antibodies, Peking, China) at 4 °C overnight. The membranes were then washed in TBST (10 mM Tris-HCl [pH 7.5], 150 mM NaCl, and 0.05% Tween-20) for 3 times (5 min each), and then incubated in a 1:3000 dilution of goat-anti-rabbit secondary antibody (bs-0295G, Bioss Antibodies, Peking, China) for 1 h at room temperature. The membrane was incubated with the chemiluminescent substrate for 2–5 min and photographed. Images were saved and digitized the gray value of each specific strip. The obtained result represented the relative content of the target protein of a sample.

### Statistical analysis

Data analysis was performed using SPSS 17.0 statistical software. The data is expressed in the form of mean ± S.D. The data obtained were multiple sets of quantitative data and analyzed by one-way analysis of variance. Firstly, the Levene test is used to test the homogeneity of variance of each group of data at each time point. The LSD-t test or the Bonferroni method was used to compare the multiples of multiple samples. The Kruskal-Wallis rank sum test is used for analysis of corneal turbidity score. *P* < 0.05 was statistically significant.

## Results

### Primary cell culture and identification of uMSCs

**Fig. 1:**
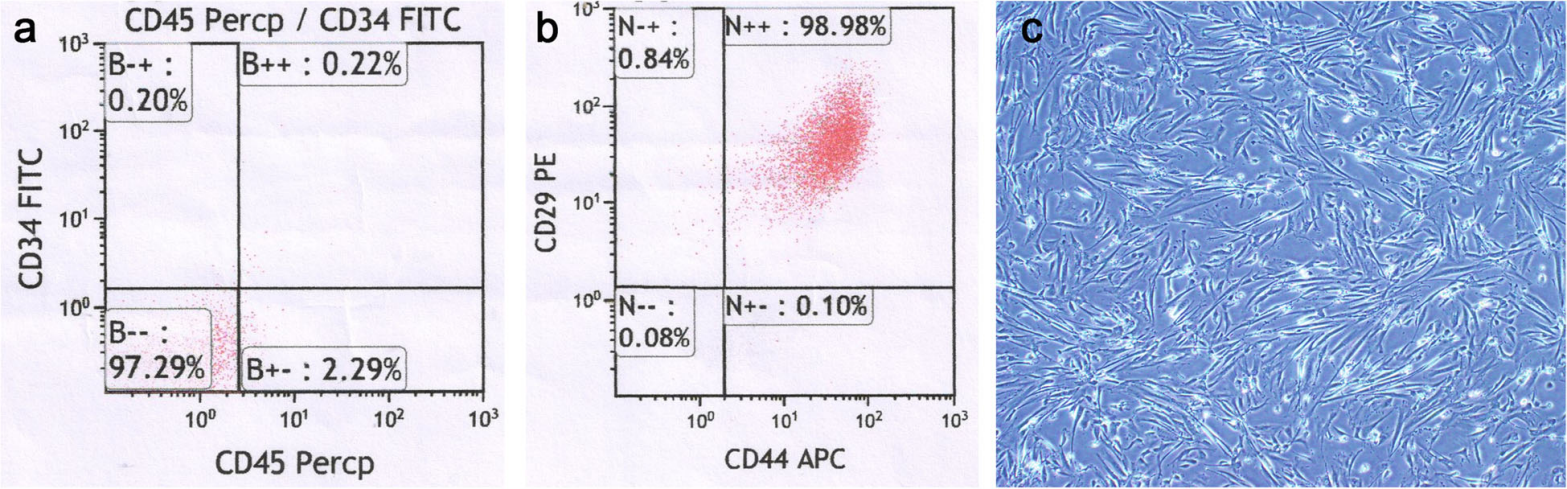
The culture and identification of human uMSCs. (**a**) Cultured cells were fluorescence labeled with CD45 and CD34. (**b**) Cultured cells were fluorescence labeled with CD29 and CD44. (**c**) The morphology of the cultured cells was characterized according to their adherence to plastic surfaces with a fibroblast-like morphology

The cultured cells were characterized according to their adherence to plastic surfaces with a fibroblast-like morphology (Fig. 1c). Flow cytometry results revealed that the cells are positive for mensenchymal stem cell surface antigen CD29 and CD44 (Fig. 1b), whereas negative for hematopoietic stem cell marker CD45 and CD34 (Fig. 1a). The number of CD45(−)CD34(−)CD29(+)CD44(+) cells accounted for 96.30% of nuclear cells.

### uMSCs alleviated corneal scar formation and enhanced slit-lamp observation and clinical score

**Fig. 2:**
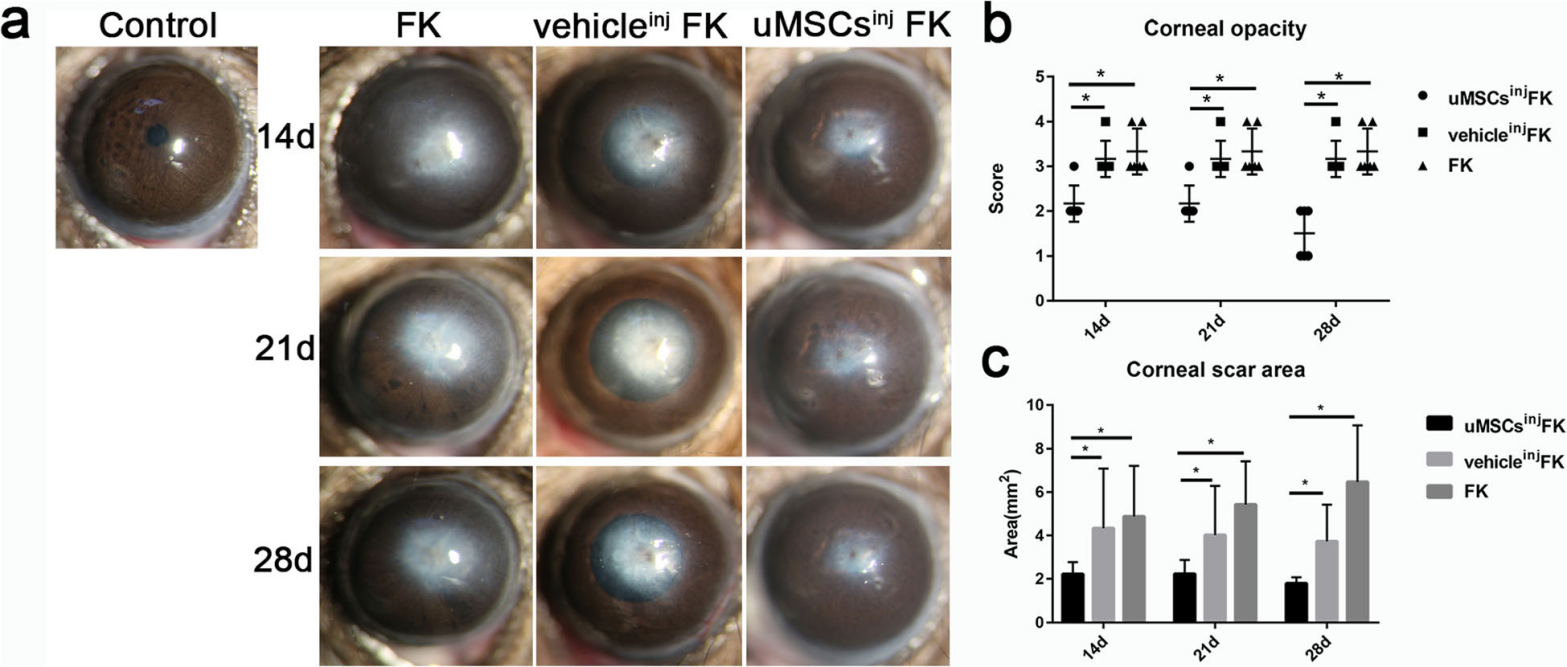
Ocular surface observation and examination. (**a**) Slit-lamp microscope observation and photograph of control group, FK group, vehicle^inj^ FK group and uMSCs^inj^ FK group. (**b**) Statistical analysis of corneal opacity scores. (**c**) Statistical analysis of corneal scar formation area (mm^2^) between groups. Magnification of ocular surface photos: 25×. Values are presented as means ± SD, *n* = 12, ^*^P < 0.05

Corneal scar formation is one of the major complications of FK and is closely related to prognosis. 12 mice of each group were involved in ocular surface examination. At 14d, 21d and 28d post-injury, uMSCs^inj^ FK group showed decreased corneal scar formation, and enhanced corneal transparency, compared with vehicle^inj^ FK group and FK group (Fig. 2a). The comparison of corneal opacity scores and scar formation area (mm^2^) between uMSCs^inj^ FK group and the other two groups were statistically significant differences both at the 14d, 21d, and 28d post-injury (Fig. 2b and c).

### Corneas from uMSCs^inj^ FK group exhibited reduced corneal thickness and inflammatory cell infiltration after FK modeling in histological examination

**Fig. 3:**
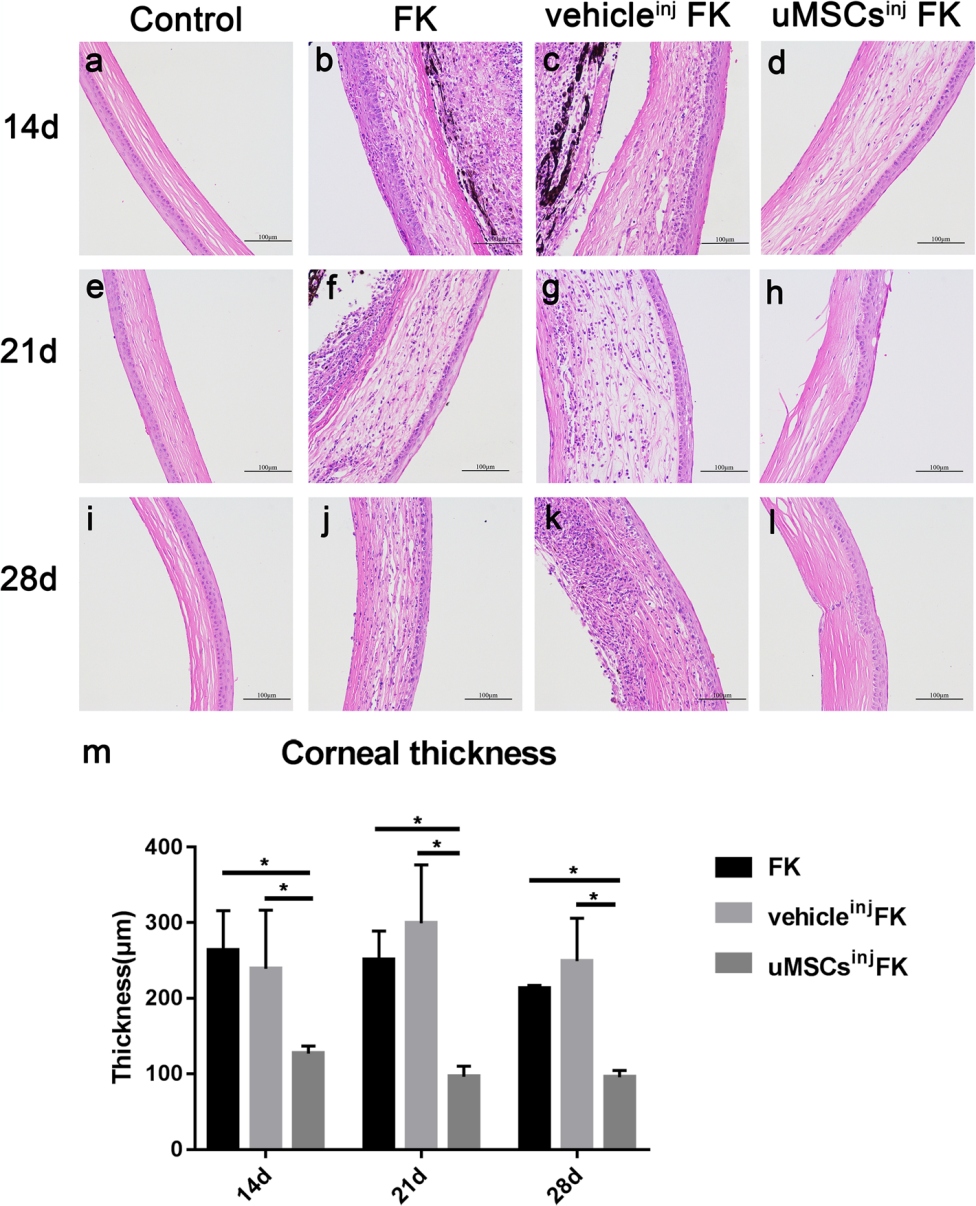
Histological examination and corneal thickness evaluation between groups. (**a**, **e** and **i**) Corneas from control group mice with no injuries. Corneas from FK group mice (**b**, **f** and **j**) and vehicle^inj^ FK group mice (**c**, **g** and **k**) exhibited corneal thickening, irregularly aligned collagen fibers and extensive inflammatory cells infiltration. (**d**, **h** and **i**) Corneas from uMSCs^inj^ FK group showed less corneal thickness, relative normal structural arrangement, and fewer inflammatory cells infiltration. (**m**) Statistical analysis of average corneal thickness (μm) between groups. Values are presented as means ± SD, *n* = 6, ^*^P < 0.05. Scale bar, 100 μm

Histological examination was performed on 14d, 21d and 28d after injury. 6 mice of each group were involved. The vehicle^inj^ FK group and FK group exhibited corneal thickening, the structure of the corneal stroma is disordered and a large amount of inflammatory cell infiltration in the whole cornea. In contrast, corneas from uMSCs^inj^ FK group showed less corneal thickness, relative normal structural arrangements, less inflammatory cell infiltration (Fig. 3a-i). The comparison of average corneal thickness (μm) between uMSCs^inj^ FK group and the other two groups had statistically significant difference at 14d, 21d and 28d post-injury (Fig. 3m).

### Collagen destruction was restored by uMSCs treatment in SHG

**Fig. 4:**
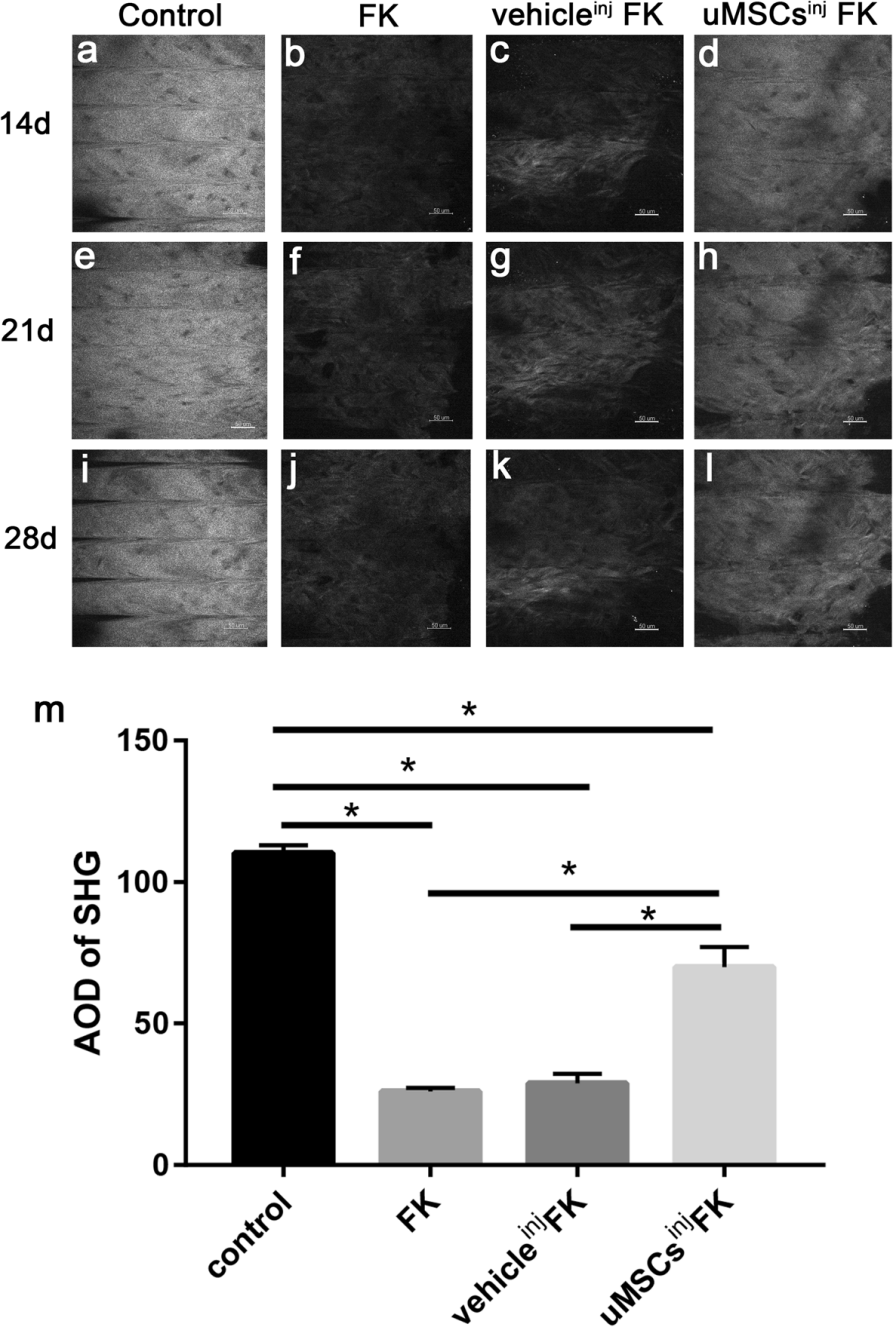
Second harmonic generation (SHG) taken by two-photon confocal microscopy in vivo. (**a**, **e** and **i**) Corneas from control group mice exhibited high intensity transient signals, indicating regular arrangement of corneal collagen fibers. Corneas from FK group (**b**, **f** and **j**) and vehicle^inj^ FK group mice (**c**, **g** and **k**) exhibited weak signals, indicating collagen degradation and disarrangement of collagen fibers caused by infection and inflammation. (**d**, **h** and **i**) The uMSCs^inj^ FK group mice corneas exhibited relative high intensity transient signals indicating relative regular collagen fibril arrangements compared with the other two groups. (**m**) Statistical analysis of average optical intensity (AOD) of SHG between groups on 14d post-injury. Values are presented as means ± SD, *n* = 6, ^*^P < 0.05. Scale bar, 50 μm

The mouse cornea is rich in collagen fibers and can generate signals under the excitation of the second harmonic of a two-photon microscope. SHG pictures were taken in vivo at 14d, 21d and 28d post-injury. 6 mice of each group were involved. The mice corneas of control group exhibited high intensity transient signals due to regular arrangement of corneal collagen fibers (Fig. 4a, e, and i). However collagen degradation caused by infection and inflammation in FK group and vehicle^inj^ FK group exhibited weak signals excited by confocal microscopy (Fig. 4b, f, and j). Of note, collagen destruction was restored by uMSCs treatment with an average optical intensity (AOD) 69.97 ± 7.09 at 14d post-injury, which had statistically significant differences compared with FK group (26.09 ± 1.27) and vehicle^inj^ FK group (28.98 ± 3.32) (Fig. 4m). Light scattering were also markedly reduced in uMSCs treatment group compared with the other two groups.

### Fibrosis-related factors were down-regulated by uMSCs administration

**Fig. 5:**
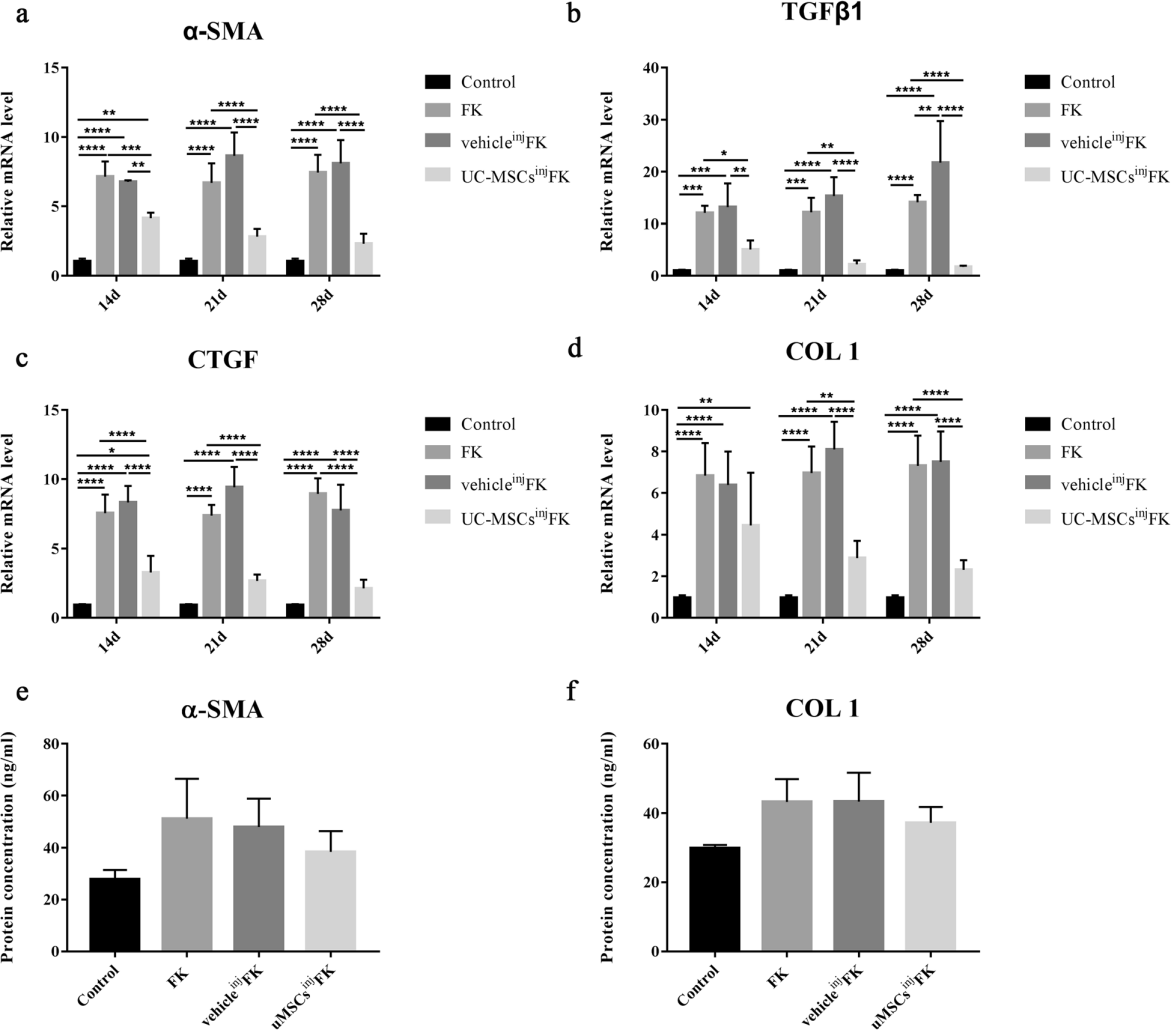
Fibrosis-related factors α-SMA, TGFβ1, CTGF and COLIwere down-regulated by uMSCs administration. (**a**-**d**) uMSCs administration significantly inhibited the expression of pro-fibrogeneic genes α-SMA, TGFβ1, CTGF and COLI and had a statistically difference compared with vehicle^inj^ FK group and FK group. No statistically significant differences were detected in the α-SMA, TGFβ1, CTGF, and COLI mRNA expression between the FK group and vehicle^inj^ FK group. (**e** and **f**) Protein concentration of α-SMA and COLIwere detected by ELISA kits at 14d post-injury, no statistical differences were found between groups. Values are given as means ± SD, *n* = 6, ^*^P < 0.05, ^**^P < 0.01, ^***^P < 0.001, ^****^P < 0.0001

Relative mRNA expression of fibrosis-related factors α-SMA, TGFβ1, CTGF and COLI in per cornea from FK group, vehicle^inj^ FK group and uMSCs^inj^ FK group were investigated by qRT-PCR at 14d, 21d and 28d post-injury. 6 mice of each group were involved. The results demonstrated that uMSCs administration significantly inhibited the expression of pro-fibrogeneic genes α-SMA (1.72-fold decrease at 14d, 2.35-fold decrease at 21d, 3.2-fold decrease at 28d; *P* < 0.05), TGFβ1 (2.37-fold decrease at 14d, 5.46-fold decrease at 21d, 8.21-fold decrease at 28d; *P* < 0.05), CTGF (2.3-fold decrease at 14d, 2.76-fold decrease at 21d, 4.18-fold decrease at 28d; *P* < 0.05) and COLI (1.53-fold decrease at 14d, 2.41-fold decrease at 21d, 3.16-fold decrease at 28d; *P* < 0.05) mRNA levels compared with vehicle^inj^ FK group (Fig. 5a-d). No statistically significant differences were detected in the α-SMA, TGFβ1, CTGF, and COLI mRNA expression between the FK group and vehicle^inj^ FK group. Protein concentration of α-SMA and COLI were detected by ELISA kits at 14d post-injury. No statistical differences were found between groups. We believe that the inconsistency of mRNA and protein detection results may be related to the low protein content in the cornea of mice.

### α-SMA production was inhibited by uMSCs administration in mice cornea after FK

**Fig. 6:**
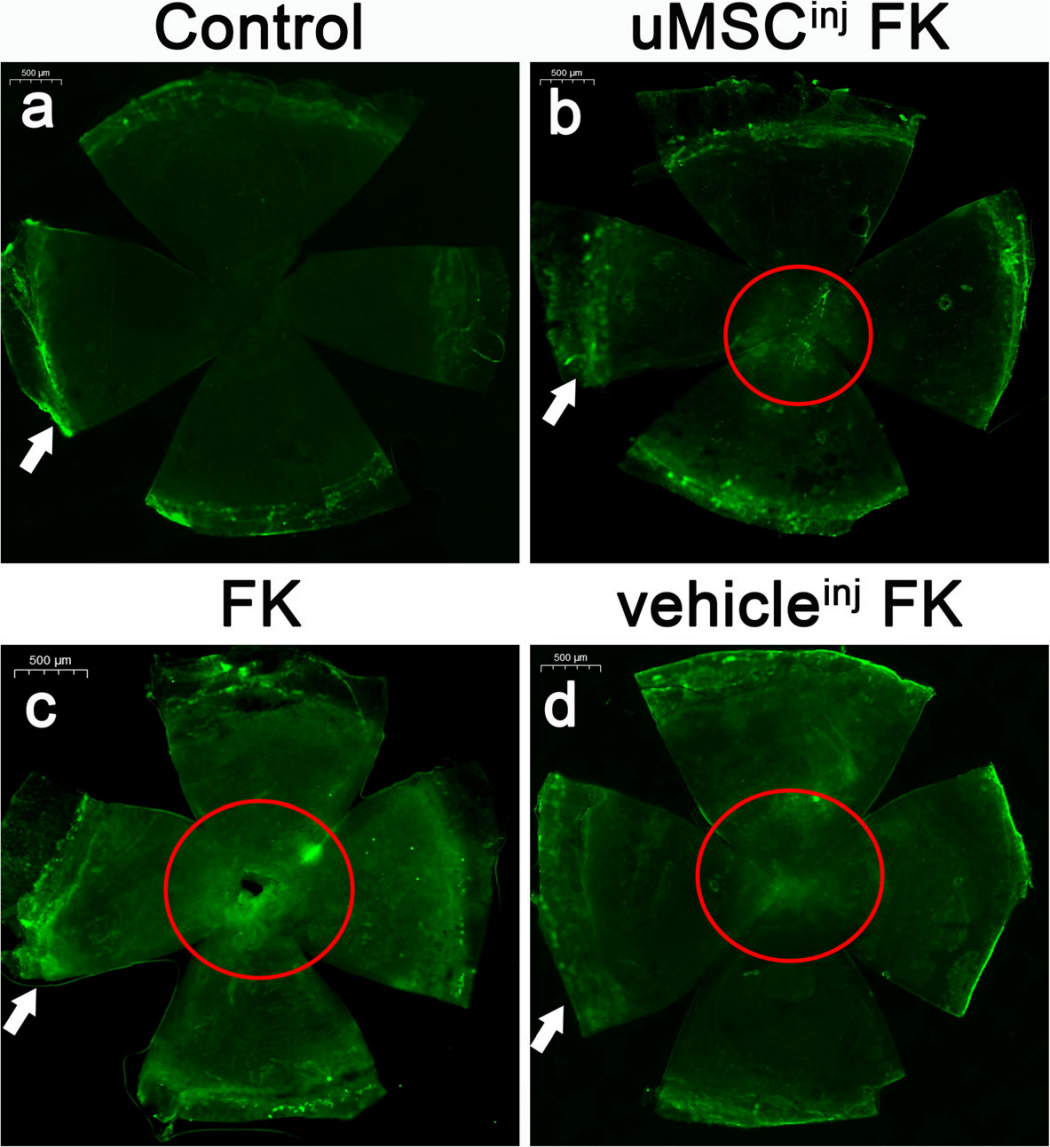
α-SMA production detected by immunofluorencence. (**a**-**d**) Immunofluorescence staining photos of corneal α-SMA expression separately from control group, uMSCs^inj^ FK group, FK group and vehicle^inj^ FK group. White arrow, α-SMA expression at the pericorneal vascular region due to vascular wall smooth muscle cells staining. Red circle, corneal lesion area. The α-SMA was labeled by FITC, *n* = 4. Scale bar, 500 μm

The α-SMA is a biochemical marker of myofibroblast. 4 mice of each group were involved. Mice corneas of control group could only be found of α-SMA expression at the pericorneal vascular region due to vascular wall smooth muscle cells staining, but was not observed in the central area of cornea (Fig. 6a). The α-SMA production was up-regulated in the lesion area of corneas in FK group and vehicle^inj^ FK group (Fig. 6c and d), whereas was found down-regulated in uMSCs^inj^ FK group (Fig. 6b), which was consistent with clinical manifestations.

### uMSCs attenuates phosphorylation of TGFβ1/Smad2 signaling pathway in the regulation of corneal fibrosis

**Fig. 7:**
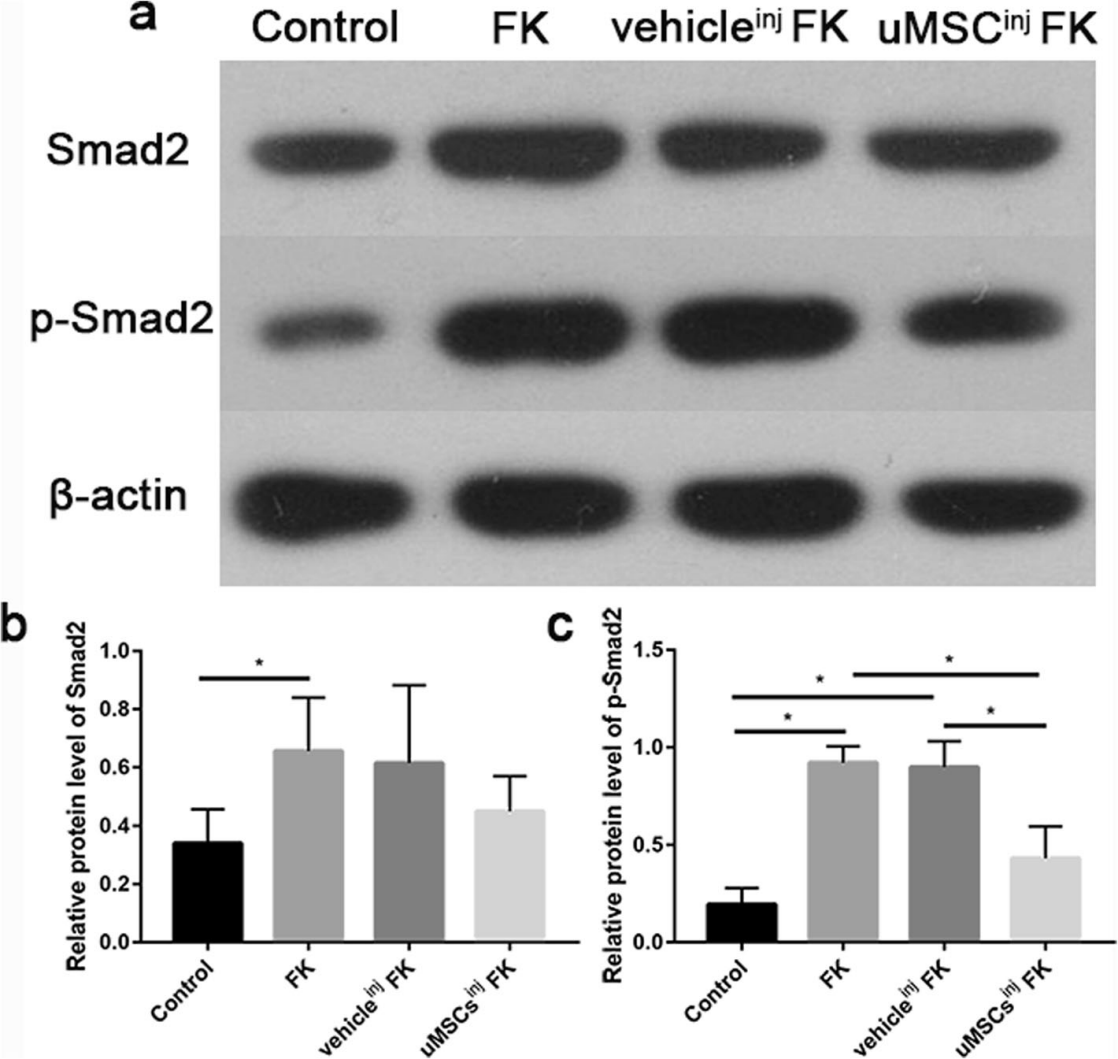
uMSCs attenuates phosphorylation of TGFβ1/Smad2 signaling pathway in the regulation of corneal fibrosis. (**a**) Westernblot analysis to detect relative protein levels of Smad2 and phosphorylated Smad2 in corneas at 14d post-injury. (**b**) Statistical analysis of relative protein expression of Smad2. (**c**) Statistical analysis of relative protein expression of phosphorylated- Smad2. Values are given as means ± SD, *n* = 6, ^*^P < 0.05

As we know, TGFβ plays an important role in the repair of corneal injury and the Smads family is one of the most important signaling pathways for TGFβ [8]. To validate the role of TGFβ1/Smad2 signaling pathway in the regulation of corneal fibrosis in mice FK model, we conducted westernblot analysis to detect relative protein levels of Smad2 and phosphorylated Smad2 in corneas at 14d post-injury (Fig. 7). 6 mice of each group were involved. Data revealed that the Smad2 expression was up-regulated in FK group compared with control group and had no statistically differences between FK group, vehicle^inj^ FK group and uMSCs^inj^ FK group (Fig. 7b). Phosphorylation of Smad2 was hard to detect in control group mice corneas whereas was up-regulated in FK group and vehicle^inj^ FK group respectively (Fig. 7c). Samples from uMSCs^inj^ FK group exhibited partially inhibited phosphorylated Smad2 expression compared with vehicle^inj^ FK group, indicating potentially down-regulation capacity of TGFβ1/Smad2 signaling pathway in FK mice corneas.

## Discussion

FK causes the formation of corneal leukoplakia. The formation of corneal leukoplakia in the late stage of FK is inseparable from the release of cytokines, activation and transformation of corneal stromal cells, destruction and remodeling of collagen structure [9–12]. Our study found that the mouse FK model can be successfully established by performing a cross scratch on the cornea and then inoculation with *Fusarium solani*. The corneal fungal infection was observed under a slit lamp microscope 24 h after wounding. Previous experiments in our research group also confirmed that the modeling method is effective [6]. The focus of this study is on the formation of corneal scars in the healing phase after FK infection control. Therefore, in the early stage of infection, natamycin eye drops antifungal treatment is carried out to kill fungi and reduce the damage of fungal growth on the cornea, thereby reducing the perforation rate of the cornea. On the basis of antifungal treatment, the rate of corneal perforation was effectively controlled (confirmed by previous experiments by our research group). The peak of FK lesion progression was 3~5d post-injury, and our eye-drop treatment continued until 7d post-injury. In this experiment, we set the observation time points to 14d, 21d and 28d post-injury, mainly to observe the changes of cornea in the early stage of scar formation. At 14 days after wounding, it was observed under the slit lamp microscope that the corneal central leukoplakia of the mouse was formed, and the pupil and iris were not visible. HE staining of pathological sections showed that the thickness of each layer of the cornea increased, the structure was disordered, and a large number of inflammatory cells infiltrated. SHG observation showed that the collagen fibers were disarranged at 14d after wounding in FK mice. Molecular examination revealed elevated expression of corneal fibrosis-associated factors α-SMA, TGFβ1, type I collagen, and CTGF in the cornea of FK mice, which is consistent with the results of Carla [13]. In addition, increased protein expression of Smad2 and phosphorylated Smad2 suggested that activation and up-regulation of the TGFβ1/Smad2 signaling pathway is accompanied by FK scar formation.

Mesenchymal Stem Cells (MSCs) treatment is widely used in ophthalmology, and its unique anti-inflammatory and immunomodulatory effects make it an ideal therapeutic mean in the treatment of dry eye, corneal epithelial damage, corneal transplantation and other diseases [14–16]. As for MSCs in restoring corneal transparency, intrastromally transplantation of uMSCs in cornea had been verified to restore the dendritic and hexagonal morphology of host keratocytes and improve corneal transparency [17]. Liu [18] revealed that corneal transparency and stromal thickness of lumican null mice were significantly improved by uMSCs transplantation. In our experiments, uMSCs were transplanted into the ocular surface of mice by subconjunctival injection at 1d, 4d, and 7d after modeling. Compared with other cell transplantation methods, subconjunctival injection can enhance the effect of uMSCs on the ocular surface, and maximize its therapeutic effect. After transplantation, it was found that there was no corneal neovascularization, uveitis and other immune rejection occurance of the experimental mice. Considering that uMSCs have low immunogenicity, the cell surface does not express MHC class II molecules, and some soluble cytokines which can inhibits lymphocyte proliferation are produced, therefore, uMSCs can induce host immune tolerance and reduce transplant rejection [19]. Our study found that uMSCs transplantation can alleviate the formation of corneal scar during healing period after FK infection control, and reduce the scar formation area and corneal turbidity. Meanwhile, the application of uMSCs could reduce corneal thickness increasing, the infiltration of inflammatory cells and destruction of collagen tissue. The expression of α-SMA, TGFβ1, CTGF and type I collagen decreased in the uMSCs^inj^FK group, and the difference was statistically significant compared with the vehicle^inj^FK group. In addition, uMSCs administration regulates the activation of the TGFβ1/Smad2 signaling pathway. Our results are supported by Fang in the skin injury model [20], and are consistent with the results obtained by Du [21] in the cornea of lumican null mice.

Compared with other available sources of MSCs, the uMSCs have the advantages of lower immunogenicity, higher capacity in proliferation, conveniently available, and less ethical controversy [20, 22]. The mechanisms involved in the therapeutic effects exerted by uMSCs, mainly focused on paracrine effects to suppress inflammation and myofibroblast differentiation. Researchers demonstrated that mRNA quantification of TSG-6 in MSCs predicted their efficacy in sterile inflammation models for corneal injury [23]. Exosomes are nanoscale membrane vesicles (30–150 nm) that transport active substances between different cells and identified as a new kind of major paracrine factor released by uMSCs [24]. Yu [25] reported that intravitreally injection of uMSCs-derived exosomes could reduce damage, inhibit apoptosis and suppress inflammation responses in laser-induced retinal injury. uMSCs-exosomal microRNAs has been found to play key roles in suppressing myofibroblast differentiation by inhibiting excess α-SMA and collagen deposition [20].

We also found some problems. It has been found that FK as an infectious keratopathy, the virulence and mechanical destruction of fungi, the infiltration of immune cells, the release of enzymes and cytokines and many other complex factors affect the prognosis of the disease. Although natamycin is used in antifungal therapy, corneal perforation is still inevitable. uMSCs is more effective in the treatment of cases without corneal perforation, while the treatment of severe inflammation and corneal perforation is not as expected. This suggests that in addition to antifungal therapy, the application of early anti-inflammatory and immunosuppressive drugs are also necessary to control the corneal scar formation in severe FK, There is no final conclusion about the mode and timing of uMSCs transplantation and relevant research need to be carried out to get the best treatment plan.

## Conclusion

The current study suggests that human uMSCs can evidently inhibit corneal inflammation and corneal fibrosis after FK wounding, the corneal opacity, scar formation area and corneal thickness can be reduced by uMSCs administration, accompanying with down-expression of α-SMA, TGFβ1, CTGF, and COLI through TGFβ1/Smad2 signaling pathway regulation. Our study has an implication for further exploration of MSCs as a novel therapy for patients with infectious eye disease and other fibrosis ocular diseases. However, further studies should be established to explore the in-depth mechanism underlying MSCs regulation in corneal fibrosis for new therapies.

## Data Availability

The datasets used and/or analyzed during the current study available from the corresponding author on reasonable request.
